# Overcoming dengue vaccine challenges through next-generation virus-like particle immunization strategies

**DOI:** 10.3389/fcimb.2025.1614805

**Published:** 2025-06-16

**Authors:** Mariana Parra-González, Lucio Nájera-Maldonado, Esperanza Peralta-Cuevas, Ashley J. Gutierrez-Onofre, Igor Garcia-Atutxa, Francisca Villanueva-Flores

**Affiliations:** ^1^ Centro de Investigación en Ciencia Aplicada y Tecnología Avanzada (CICATA), Unidad Morelos del Instituto Politécnico Nacional (IPN), Xochitepec, Mexico; ^2^ Computer Science Department, Universidad Católica de Murcia (UCAM), Murcia, Spain

**Keywords:** dengue virus, virus-like particle, vaccine, antibody-dependent enhancement (ADE), immunity

## Abstract

Dengue fever represents an escalating global health threat, as unprecedented outbreaks expose significant limitations of current vaccine strategies. Conventional live-attenuated dengue vaccines, while partially efficacious, face critical hurdles including serotype imbalances and antibody-dependent enhancement (ADE). This review critically assesses virus-like particle (VLP) vaccines as a promising alternative, providing safer, non-replicating platforms that mimic viral structure without risks associated with live replication. Technological advancements in recombinant expression systems have improved VLP yield, stability, and scalability, addressing deployment obstacles. Recent preclinical studies demonstrate that tetravalent dengue VLP vaccines induce balanced neutralizing antibodies across all serotypes, effectively circumventing ADE in animal models. These findings suggest superior safety and robust immune responses, potentially surpassing live-attenuated and mRNA-based vaccines. We emphasize advancements in VLP vaccine technology, including novel tetravalent particle designs engineered to exclude ADE-related immunopathogenic components (prM protein), innovative stability-enhancing formulation techniques, and cost-effective recombinant production platforms (yeast and plant-based systems). Additionally, this review proposes novel deployment strategies, such as regional manufacturing hubs, standardized modular VLP platforms, adaptive clinical trial frameworks leveraging surrogate endpoints, and strengthened international coordination for equitable vaccine distribution. Integrating these scientific innovations and practical strategies positions dengue VLP vaccines as pivotal next-generation solutions for global dengue prevention.

## Graphical Abstract

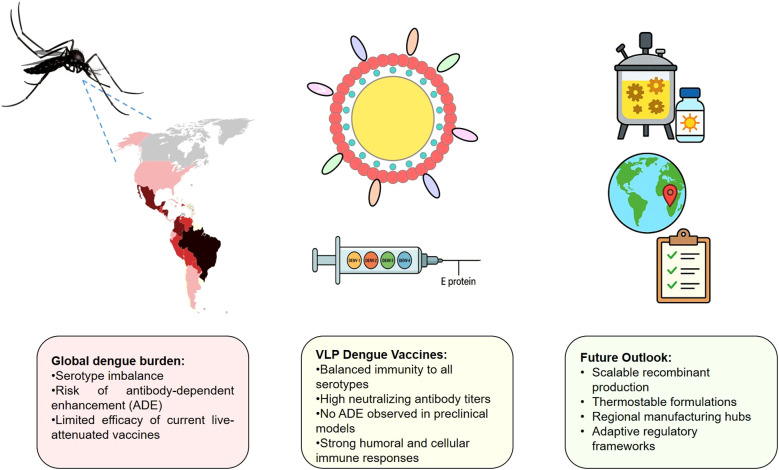

## Highlights

- This review critically evaluates the potential of virus-like particle (VLP)-based vaccines for dengue, highlighting their inherent safety as non-replicating platforms with authentic antigenic structures.
- Recent preclinical studies are synthesized and analyzed, demonstrating that tetravalent VLP vaccines induce robust, balanced neutralizing antibody responses against all four dengue serotypes, significantly minimizing the risk of antibody-dependent enhancement (ADE).
- Innovative recombinant production platforms for VLP vaccines, particularly yeast and plant-based systems, are discussed and compared, emphasizing their scalability, reduced costs, and practical applicability for global manufacturing.
- An in-depth analysis is provided on advancements in stability and yield of VLP vaccines, addressing critical limitations through optimized culture conditions, advanced purification methods, and novel formulations that substantially enhance long-term stability.
- Novel deployment strategies are proposed, including establishing regional manufacturing hubs in endemic regions and standardizing modular VLP production platforms, which would facilitate regulatory approval, vaccine accessibility, and efficient global deployment.
- Adaptive regulatory frameworks and innovative international financing mechanisms, including validated immunological correlates of protection, are recommended to accelerate clinical translation and ensure equitable global distribution of dengue VLP vaccines.

## Introduction

Dengue fever has re-emerged as a critical global public health threat, with roughly half of the world’s population now living in dengue-endemic regions​ (Thoresen et al., 2024). The global incidence of dengue has increased more than eightfold over the past two decades, primarily driven by rapid urbanization, climate change, globalization, and insufficient vector-control strategies (Stanaway et al., 2016; Messina et al., 2019). Dengue virus (DENV) infections are surging to unprecedented levels; in 2023 alone, over 6.5 million cases and approximately 6,800 deaths were reported worldwide, the highest annual totals on record​ (Haider et al., 2024). Recent outbreaks have notably affected the Americas and Southeast Asia, with South America accounting for nearly 4 million cases in 2023. This escalating disease burden emphasizes the urgent need for effective dengue control measures, including improved vaccines, given that conventional vector control has proven insufficient and no specific antiviral therapy is available (Laydon et al., 2021; Haider et al., 2024).

**Figure 1:**
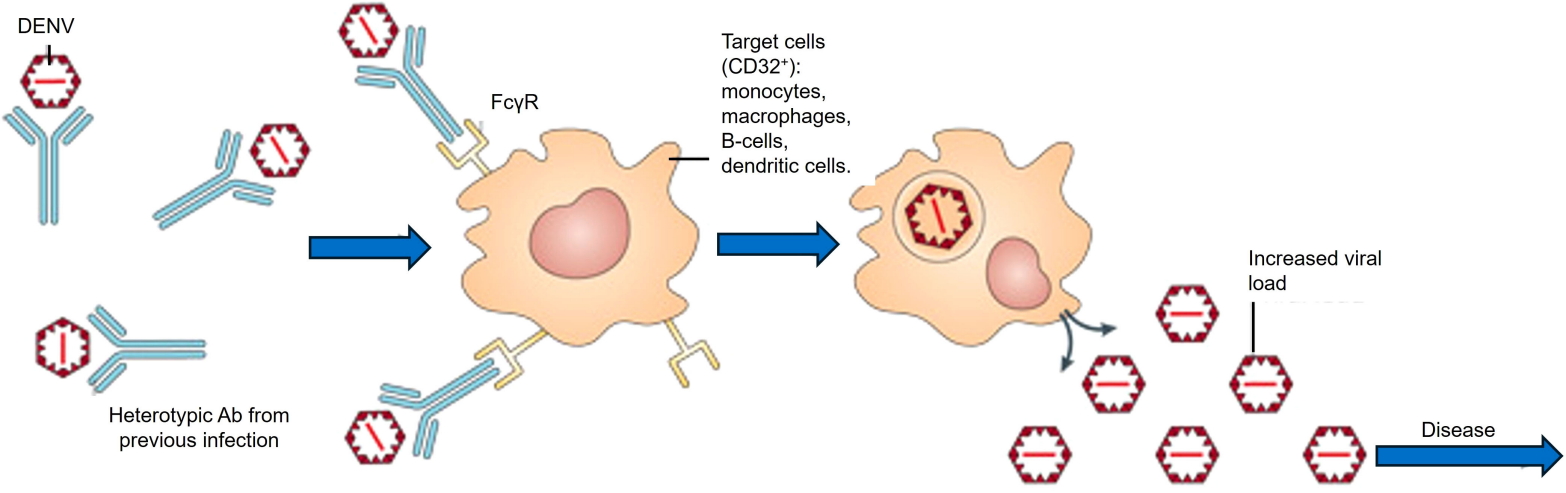
Mechanism of ADE in DENV infection. Pre-existing heterotypic antibodies from a previous dengue infection bind to DENV particles without effectively neutralizing them. These antibody-virus complexes subsequently attach to Fc-gamma receptors (FcγR) expressed on immune cells such as monocytes, macrophages, B-cells, and dendritic cells, facilitating viral entry, enhancing intracellular replication, and consequently increasing viral load and disease severity.

However, developing a safe and broadly efficacious dengue vaccine presents unique challenges. DENV comprises four antigenically distinct serotypes, and immunity acquired against one serotype confers only transient cross-protection against the others (Thoresen et al., 2024). This temporary cross-protection arises from serotype-specific antibody responses unable to broadly neutralize other serotypes, increasing the risk of severe disease due to antibody-dependent enhancement (ADE). ADE occurs when pre-existing, sub-neutralizing antibodies bind heterotypic DENV particles, facilitating enhanced viral entry into Fcγ receptor-expressing immune cells, causing higher viral replication and intensified inflammation that significantly contributes to severe dengue (Guzman and Vazquez, 2010) (**Figure 1**). Achieving such tetravalent immunity has proven difficult. The first licensed dengue vaccine, Sanofi’s CYD-TDV (Dengvaxia), showed only moderate efficacy (around 56–61% in phase III trials)​ (Laydon et al., 2021). Long-term follow-up revealed an elevated risk of severe dengue hospitalizations in vaccine recipients without prior dengue exposure, restricting Dengvaxia to seropositive populations. Newer live-attenuated vaccines have demonstrated improved efficacy, such as Takeda’s tetravalent candidate (TAK-003, recently branded Qdenga^®^). In a phase 3 trial, TAK-003 achieved approximately 61% overall efficacy (and ~84% efficacy against hospitalization) over 4.5 years of follow-up​ (Tricou et al., 2024). Nonetheless, vaccine construct differences, particularly viral attenuation methods and antigen presentation, continue to significantly influence both efficacy and ADE risk significantly, underscoring ongoing challenges in dengue vaccine development (Wilder-Smith, 2020). These limitations highlight the continuing hurdles in dengue vaccine development: ensuring safety (avoiding ADE) and comprehensive protection against all serotypes (Thoresen et al., 2024).

Virus-like particle (VLP) vaccines have emerged as an innovative approach to meet these challenges. VLPs are self-assembling structures composed of viral structural proteins that closely mimic the morphology and antigenic configuration of the native virus but contain no viral genome. By presenting DENV envelope proteins in their authentic conformations on a particle surface, VLPs display the repetitive, high-density epitopes needed to trigger potent neutralizing antibody responses. Because VLPs lack genetic material, they are non-infectious and intrinsically safer than live vaccines. This is particularly advantageous for young children and immunocompromised individuals at high risk of dengue. VLP-based vaccines have an established track record in other viral diseases (e.g., hepatitis B and human papillomavirus) that elicit robust immunity with excellent safety. Recent preclinical studies using murine and primate models confirm that dengue-specific VLPs induce protective neutralizing antibodies without evidence of ADE (Thoresen et al., 2024).

Another critical aspect of VLP vaccine development is the production and stability of the particle formulations. Dengue VLPs are typically produced via recombinant expression systems (in insect, mammalian, or even plant cells) and must self-assemble correctly to maintain the native epitopes necessary for immunity. Early studies indicated that achieving high yield and stability for flavivirus VLPs could be challenging, but recent technological advances address these issues. For instance, Fan et al. (2024) demonstrated that optimizing cell culture conditions (a temperature-shift protocol) can dramatically increase flavivirus VLP yields, consistently exceeding 1 μg/mL for dengue VLPs, without altering the conformational integrity of the envelope protein epitopes (Fan et al., 2024). Other recent strategies, such as advanced downstream purification methods and nanotechnology-based formulations, have also shown promising improvements in yield, purity, and stability of dengue VLP vaccines (Vicente et al., 2011). Such bioprocessing and formulation stability improvements are pivotal for making VLP vaccines practically viable, ensuring that the final product remains immunogenic over time and under field conditions (e.g., during storage and transport in tropical climates). These advances pave the way for scaling up dengue VLP vaccine production and evaluating their performance in clinical settings.

In the following sections, we critically examine the latest progress in dengue VLP vaccine research, focusing on vaccines against dengue challenges, methods for VLP production, strategies to enhance their stability, and the quality of the immune responses they provoke. We evaluate these aspects based on defined criteria, including immunogenicity, efficacy, safety profiles, production scalability, and field stability. We synthesize evidence from recent studies to assess how VLP-based dengue vaccines are overcoming previous limitations and to what extent they fulfill the requirements of safety and broad efficacy. By highlighting these developments, this review aims to clarify the potential of VLP vaccines as next-generation candidates against dengue and related flavivirus diseases and to identify remaining challenges on the path toward an effective dengue vaccine.

## Dengue virus: current vaccine development challenges

**Figure 2:**
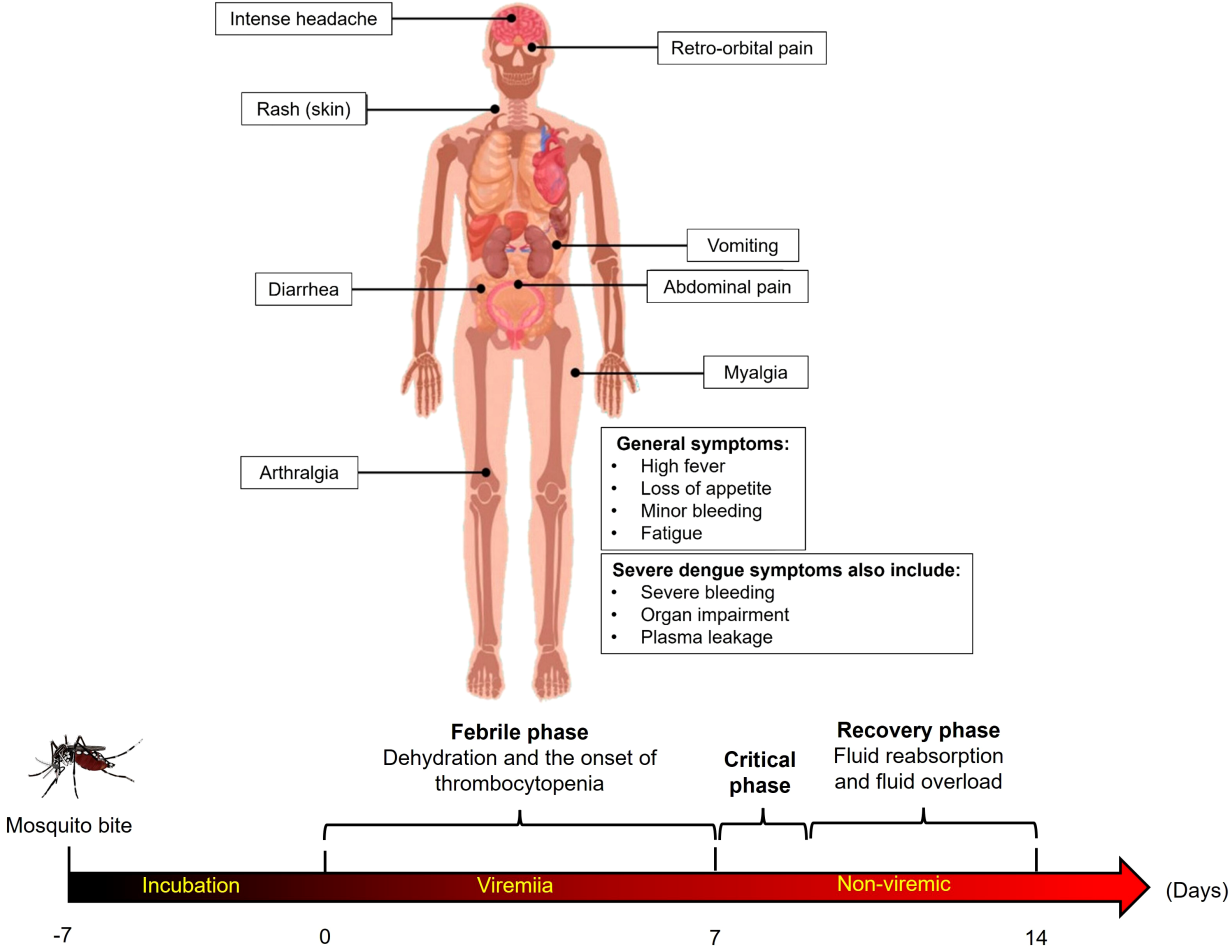
Schematic representation of the primary clinical manifestations of hemorrhagic dengue. Common symptoms include high fever, intense headache, retro-orbital pain, skin rash, myalgia, arthralgia, gastrointestinal symptoms (vomiting, diarrhea, and abdominal pain), and minor bleeding. Severe cases also present severe bleeding, plasma leakage, and organ impairment, indicating progression to life-threatening complications.

Despite considerable progress in recent years, dengue vaccine development faces several platform-specific challenges. The first licensed vaccine, CYD-TDV (Dengvaxia), based on a yellow fever 17D virus backbone expressing dengue structural proteins, demonstrated uneven protection across dengue serotypes, particularly low efficacy against DENV-2 (~35%) (Ferguson et al., 2016; Flasche et al., 2016; Laydon et al., 2021; Pintado Silva and Fernandez-Sesma, 2023). **Figure 2** presents a schematic representation illustrating the primary clinical manifestations of hemorrhagic dengue.

Follow-up studies further identified safety concerns associated with increased hospitalization risk among seronegative vaccine recipients, restricting its practical use to previously dengue-exposed populations and complicating public health implementation (Hadinegoro et al., 2015; Laydon et al., 2021). Subsequently developed live-attenuated vaccines, such as TAK-003 (Qdenga^®^), have improved initial efficacy (~80%) and show substantial protection against dengue-associated hospitalizations. Nonetheless, efficacy remains variable across serotypes and decreases notably after two years, raising concerns about long-term durability (Biswal et al., 2020; López-Medina et al., 2022; Pintado Silva and Fernandez-Sesma, 2023).

Emerging vaccine technologies, including virus-like particles (VLPs), purified protein subunits, and novel mRNA-based approaches, seek to overcome these limitations. However, these platforms face distinct technical hurdles related to consistent antigen presentation, particle stability, scalable production, and robust immunogenicity in diverse human populations (Urakami et al., 2017; Rothen et al., 2024). The following sections critically evaluate these emerging dengue vaccine platforms, emphasizing their potential, unique challenges, and pathways toward practical deployment.

## VLPs as a promising platform for dengue vaccine development

**Figure 3:**
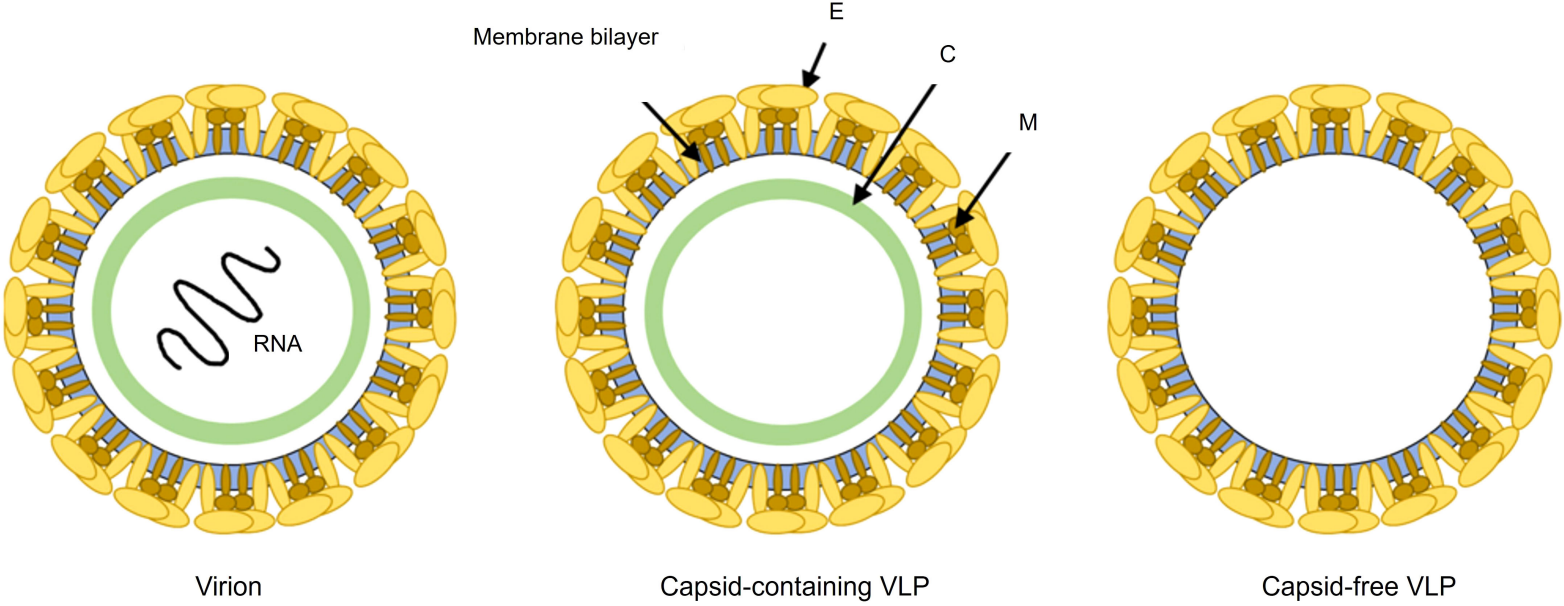
Schematic representation comparing dengue virion and VLPs. The infectious virion contains structural proteins: envelope (E), membrane (M), and capsid (C), encapsulating viral RNA. VLPs, either capsid-containing or capsid-free, mimic the authentic viral structure but lack viral RNA, rendering them non-infectious. The preservation of native envelope protein (E) epitopes, the absence of genetic material, and the inability to replicate make VLPs an ideal and safe platform for dengue vaccine development. The timeline illustrates the typical disease phases: incubation (−7 to 0 days post-infection), viremia and febrile phase (0–7 days), critical phase with highest risk of severe complications (~day 7), and the non-viremic recovery phase (7–14 days).

Given the limitations of current dengue vaccines, VLPs have emerged as a promising next-generation platform for vaccine development. VLPs closely mimic the structural and antigenic properties of native dengue virions but lack the viral genome, significantly enhancing their safety (**Figure 3**). Moreover, their flexible design allows precise antigen presentation, potentially overcoming serotype-specific immunity gaps and considerably reducing the ADE risk by excluding specific immunopathogenic elements such as the prM protein (De Alwis et al., 2014). Anti-prM antibodies are highly cross-reactive and poorly neutralizing; they can even render immature virions infectious by mediating Fcγ receptor-dependent uptake (Dejnirattisai et al., 2010; Rodenhuis-Zybert et al., 2010). By omitting prM, VLP immunogens focus the immune response on the envelope (E) glycoproteins and avoid this problematic antibody subpopulation. Moreover, the VLPs present the E proteins in their native, virion-like conformation (i.e., as correctly folded dimers on a particulate surface), preserving critical quaternary neutralizing epitopes (Rouvinski et al., 2015). This structural integrity promotes the elicitation of strongly neutralizing antibodies targeting conformational epitopes (rather than the immunodominant but less protective fusion loop or other cross-reactive regions), thereby skewing the antibody repertoire away from serotype-cross-reactive, non-neutralizing specificities. Consistent with these mechanisms, fully mature dengue VLPs elicited significantly lower levels of enhancing anti-prM antibodies and higher neutralizing antibody titers than immature particle immunogens in experimental models, supporting the notion that VLP vaccines inherently bias the response toward protective immunity (Shen et al., 2018).

The antibody profile induced by dengue VLP vaccination consequently shows a diminished capacity to mediate ADE. In contrast to certain live or subunit immunizations, polyclonal sera from VLP-vaccinated animals have demonstrated no *in vitro* enhancement of DENV infection in FcγR-expressing cells. This outcome reflects the dominance of potent neutralizing antibodies: robust neutralization prevents the formation of subneutralizing antibody–virus complexes, precluding the efficient FcγR-mediated entry that underlies ADE (Urakami et al., 2017). Additionally, qualitative differences in the Fc region of VLP-elicited antibodies may contribute to reduced ADE potential. Severe dengue disease has been linked to antibodies with robust FcγR affinity (for example, afucosylated IgG1), underscoring that Fc–FcγR interactions are a key driver of ADE pathology (Bournazos et al., 2021). Notably, non-replicating immunogens like VLPs do not tend to induce the skewed afucosylated IgG response observed in natural infection or live-virus vaccination (Larsen et al., 2021). Thus, antibodies generated by VLP vaccines are less likely to engage Fcγ receptors in a manner that enhances infection, instead favoring classical neutralization and immune clearance pathways. Together, the exclusion of prM, maintenance of native E protein structure, and an immunoglobulin response less prone to pathological FcγR engagement all contribute to the markedly lower risk of ADE with dengue VLP vaccines.

On the other hand, VLPs are self-assembling, multi-protein nanoparticles that closely resemble authentic virus particles in structure and antigenic composition, but are non-infectious​ (Rothen et al., 2024; Abbo et al., 2025). By expressing viral structural proteins (such as capsid or envelope components) in suitable *in vitro* systems, these proteins spontaneously assemble into particles mimicking native virions without genetic material, thereby preventing viral replication or pathogenic reversion. VLPs maintain authentic viral surface protein conformations, including complex quaternary epitopes, ensuring accurate immune-recognition antigenicity (Rothen et al., 2024).

Despite lacking a genome, VLPs are highly immunogenic due to their multivalent, densely repetitive antigenic arrays. This ordered display enables efficient cross-linking of B-cell receptors, strongly stimulating B-cell activation​ (Hua and Hou, 2013; Brune et al., 2016). Additionally, the nanoparticle size (20–200 nm) of VLPs promotes efficient lymph node drainage and antigen-presenting cell uptake, amplifying immune responses compared to soluble antigens or traditional subunit vaccines (Bachmann and Jennings, 2010). VLP preparations can also contain inherent adjuvant properties, such as encapsidated RNA engaging innate immune receptors like Toll-like receptors, further enhancing immunogenicity​ (Hua and Hou, 2013; Zabel et al., 2013). Thus, VLPs achieve an optimal balance between immunogenic potency and safety, making them ideal candidates for novel vaccine platforms.

Several licensed human vaccines demonstrate the utility of VLP technology. The hepatitis B vaccine, composed of HBV surface antigen (HBsAg) VLPs introduced in 1986, drastically reduced global HBV incidence, validating the safety and efficacy of this platform (Mohsen et al., 2017). Similarly, HPV vaccines based on L1 protein VLPs have demonstrated exceptional clinical efficacy, effectively preventing HPV-induced cervical lesions (The FUTURE II Study Group, 2007). Other successful examples include hepatitis E and malaria (RTS,S) vaccines, underscoring VLP platforms’ broad applicability and established regulatory acceptance (Cao et al., 2018; Marini et al., 2019).

Given these successes, VLPs offer substantial promise for dengue vaccine development, especially considering dengue’s distinct challenges, including serotype diversity and ADE risk. Tetravalent dengue VLP candidates, displaying correctly folded envelope proteins, are being actively explored to elicit balanced neutralizing responses against all serotypes without ADE risks (Rothen et al., 2024). Recent preclinical studies have shown promising results, such as balanced neutralizing antibody titers against all four dengue serotypes in animal models without triggering ADE, supporting the viability of this approach. However, significant technical challenges remain, including ensuring consistent particle assembly, maintaining antigenic stability during storage and transport, and efficiently scaling up production (Metz et al., 2015; Urakami et al., 2017). Additionally, economic factors and regulatory complexities observed with previously licensed VLP vaccines (e.g., HPV and malaria) highlight that cost-effective production methods, rigorous quality control, and streamlined regulatory pathways will be critical determinants for successfully deploying dengue VLP vaccines globally (Lua et al., 2014; Mohsen et al., 2017). The feasibility and scalability of VLP vaccines also heavily depend on choosing appropriate expression systems, which is critical for effective mass production. The following section discusses various production platforms for dengue VLP vaccine development.

## Production platforms for VLPs for dengue vaccine development

**Figure 4:**
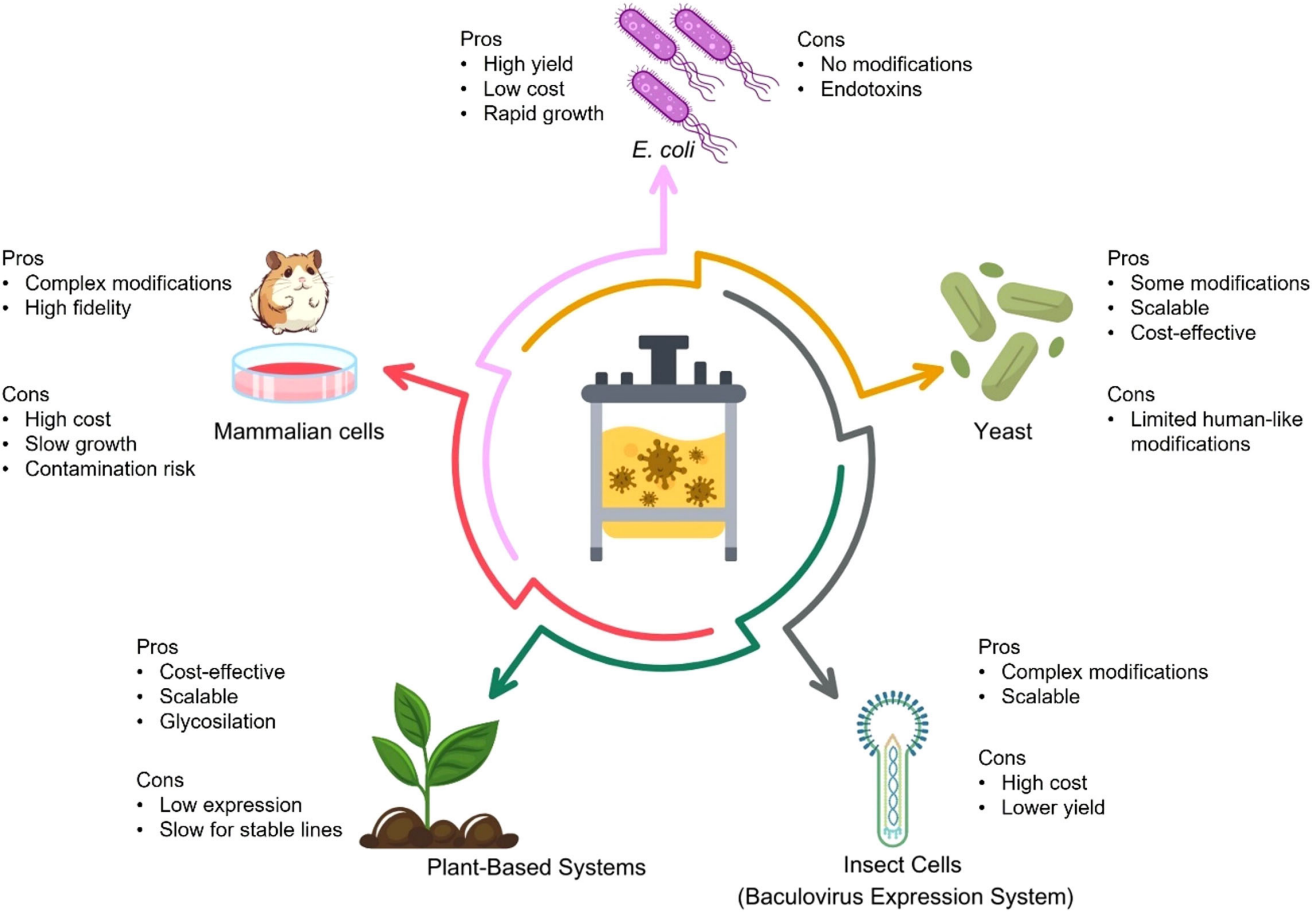
Overview of expression systems used for VLP) production.Different recombinant platforms, including *E. coli*, yeast, insect cells (baculovirus expression system), mammalian cells, and plant-based systems, offer distinct advantages and limitations for VLP vaccine development. While *E. coli* enables rapid and high-yield expression at low cost, it lacks post-translational modifications and poses endotoxin risks. Yeast systems are cost-effective and scalable, with limited human-like glycosylation. Insect cells provide complex modifications and scalability, but at a higher cost and lower yield. Mammalian cells offer the most authentic protein processing but are costly and slower growing. Plant-based systems are scalable and safe but limited by slower expression and lower yields. Selecting the optimal platform involves balancing cost, scalability, fidelity of antigen presentation, and downstream processing needs.

Selecting an appropriate production platform is crucial for successfully developing and implementing VLP vaccines against the DENV. The following section outlines key characteristics, advantages, and disadvantages of various recombinant systems used in virus-like particle production (**Figure 4**).

### Mammalian cell systems (e.g., HEK293, CHO)

Mammalian cell systems (CHO, HEK293) provide authentic post-translational modifications (PTMs), particularly complex glycosylation patterns essential for eliciting potent and broadly neutralizing antibody responses. Transient expression in HEK293 cells typically yields ~5–10 mg/L of purified dengue VLP antigen, even with yield-enhancing mutations (Thoresen et al., 2024). Although stable CHO cell processes can achieve up to ~1 g/L under optimized conditions, mammalian platforms generally produce moderate yields at higher production costs due to expensive media and lower-density culture conditions (Dai et al., 2018). However, glycosylation fidelity in mammalian systems approaches 100% human-like, providing antigenic epitopes similar to native virions. Despite moderate yields and higher associated costs, mammalian-produced VLP vaccines are well-established in regulatory contexts, exemplified by recently licensed enveloped vaccines (Fan et al., 2024; Sanchez-Martinez et al., 2024).

### Insect cell systems (e.g., Sf9 with Baculovirus)

Insect cell platforms (typically Sf9 or High Five cells infected with a baculovirus vector) are widely used for VLP production and offer faster, higher-density culture than mammalian cells. In many reports, yields for dengue VLPs in insect cells are moderate, on the order of single-digit mg/L after purification. For example, a tetravalent dengue VLP produced in insect cells (with prM/E co-expression) reported purified yields up to ~3 mg/L for DENV-3 and ~1.5 mg/L for DENV-1 VLPs (Urakami et al., 2017). Similarly, other studies note that secreted glycoprotein yields in baculovirus systems are often low, partly due to baculovirus-induced stress on the secretory pathway (Dai et al., 2018). With process optimization (e.g., using strong polyhedrin promoters, or lower-temperature fed-batch cultures), higher titers can be achieved for simpler VLPs, e.g., HPV VLP yields of ~60 mg/L in Sf9 cells have been reported (Wang et al., 2022). However, such high titers are more challenging for the complex, enveloped dengue VLPs. Production costs in insect cell systems are moderate, significantly lower than in mammalian systems. The baculovirus platform benefits from cheaper media and fewer biosafety concerns, allowing relatively economical scale-up (for instance, influenza VLP vaccines produced in insect cells have the cost of goods comparable to egg-based processes) (Dai et al., 2018). Glycosylation fidelity in insect-derived dengue VLPs is incomplete. Lepidopteran cells lack certain enzymes, so N-linked glycans remain predominantly high-mannose type (with little to no terminal sialylation or complex branching) (Yap et al., 2017). This means dengue E glycoproteins from insect cells carry under-processed glycans (similar to those on virions from mosquito cells). While this altered glycosylation can influence antigenicity and immunogenicity, it may not severely impair the immune response. Studies have shown that insect-cell dengue VLPs can still induce neutralizing antibodies, though sometimes at lower titers than their mammalian-cell counterparts (Abbo et al., 2023). These systems are widely accepted in regulatory frameworks, as evidenced by licensed vaccines against HPV, influenza, and SARS-CoV-2, underscoring their suitability for dengue VLP vaccine production (Trombetta et al., 2022; Utomo et al., 2022; Hong et al., 2023). Overall, insect platforms offer a good compromise of yield and cost but require attention to glycosylation differences and optimization of secretion to maximize VLP recovery.

### Yeast systems (e.g., *Pichia pastoris*)

Yeast expression systems have emerged as a promising, high-yield, low-cost platform for dengue VLP production. *P. pastoris* (now *Komagataella phaffii*) can grow to very high cell densities in inexpensive defined media, enabling high volumetric productivity. Although enveloped VLP assembly in yeast can be challenging (yeast do not naturally secrete flavivirus particles, so that VLPs may accumulate intracellularly), several groups have successfully produced dengue VLPs or VLP-based subunits in yeast. Yields in yeast are reported to be competitive with or even higher than insect cell systems. Yeast expression of a similar enveloped alphavirus VLP (Chikungunya) reached ~60 mg/L, which was ~3-fold higher than the yield in insect cells and ~1.5-fold higher than in mammalian cells for that antigen (Thompson et al., 2022). While specific yields for dengue VLPs in yeast vary, one example is a dengue-2 prM/E construct under a constitutive *Pichia* promoter that produced high levels of E antigen; electron microscopy confirmed abundant VLPs in both cells and supernatant (Liu et al., 2010). With fermentation scale-up, it is reasonable to expect dengue VLP titers on tens of mg/L in yeast. The production cost in yeast is very low, since media (often just glycerol/methanol, minerals) is cheap and the process is simple, batch or fed-batch fermentation. Yeast is a GRAS organism and well-suited for cost-sensitive vaccines. Indeed, *P. pastoris* is highlighted as a cost-effective tool for LMIC vaccines, including dengue (Tripathi et al., 2015). Glycosylation fidelity in yeast is a drawback relative to higher eukaryotic systems; yeast adds high-mannose N-glycans that differ from human types. *P. pastoris* N-glycans are shorter than those in S. cerevisiae but are still mainly mannose-rich (and lack mammalian complex sugar residues). Dengue E protein expressed in *P. pastoris* is glycosylated (both Asn-67 and Asn-153 occupied), and these glycans can even be enzymatically trimmed to investigate their role (Tripathi et al., 2015). While not human-like, yeast glycoforms have been tolerated in animal models: *Pichia*-made DENV VLPs elicited neutralizing antibodies in mice (De Sá Magalhães and Keshavarz-Moore, 2021). Ongoing engineering efforts (humanized glycosylation pathways in yeast) may further improve fidelity. In summary, yeast platforms can deliver high yields and low costs for dengue VLPs, with the trade-off of non-human glycosylation that must be evaluated for its impact on vaccine efficacy.

### Plant-based expression (transient in *Nicotiana* spp.)

Plant molecular farming is a novel platform tested for dengue VLP production. Using transient Agroinfiltration in *Nicotiana benthamiana*, researchers have co-expressed DENV structural proteins (prM and E, sometimes with supporting viral proteases or chaperones) to assemble VLPs in plant cells. A key advantage of plant systems is very low production cost; plants can be grown at scale in greenhouses or fields with minimal infrastructure, and there is no risk of human pathogen contamination (Dai et al., 2018; Peralta-Cuevas et al., 2025). Plants also perform eukaryotic post-translational modifications, although these are plant-specific. Reported yields of dengue VLPs in plants are currently the lowest of all platforms, highlighting the technical challenges. Ponndorf et al. (2021) produced DENV-1 VLPs in *N. benthamiana* and, after optimizing constructs and purification, obtained only ~2 µg of VLP per gram of fresh leaf tissue. Despite robust transient expression systems, this is equivalent to <0.01 mg/L of plant extract (a very low expression level). The authors noted that such yields are much lower than yields for non-enveloped VLPs in plants, which can reach tens of µg/g or higher. For example, bluetongue virus VLPs (non-enveloped core-like particles) went 5–15 µg/g in the same plant system. The low dengue VLP yield is likely due to inefficient assembly and secretion in plant cells, as many particles remain bound to membranes or aggregated with impurities, requiring multiple ultracentrifugation steps (Ponndorf et al., 2021). Scalability of plant production is theoretically high (hectares of plants could be used), but with current yields, enormous biomass would be required per dose. Glycosylation fidelity is another consideration: plants add N-glycans with core α-1,3-fucose and β-1,2-xylose, distinct from human glycans and can be antigenic in humans (Dai et al., 2018). These improper glycoforms could reduce vaccine efficacy or cause adverse immune reactions. Efforts are underway to engineer glyco-optimized plant lines or to remodel plant glycans on VLPs enzymatically. Despite these challenges, plant-produced dengue VLPs have shown immunogenicity in mice (Ponndorf et al., 2021; Mardanova et al., 2024). The plant platform remains attractive for its rapid, scalable output and low cost, but significant optimization is needed to approach the yield and consistency levels of more established systems.

Selecting the optimal production platform depends on yield, glycosylation fidelity, and subsequent considerations like vaccine stability and formulation. **Table 1** summarizes the main characteristics of different expression platforms for dengue VLP vaccine production, highlighting key aspects such as yield, glycosylation patterns, scalability, regulatory acceptance, and production costs. This comparative overview aids in identifying suitable production platforms for dengue VLP vaccines.

**Table 1:** Comparison of expression platforms for dengue VLP vaccine.

Platform	Expression systems	Typical yield	Glycosylation and PTMs	Scalability and cost	Regulatory track record and examples	Feasibility for dengue VLP vaccines	Production costs	References
Mammalian Cells	CHO, HEK293	Moderate (~1 mg/L)	Human-like glycosylation; authentic PTMs, including complex glycans	Highly scalable, expensive media, strict aseptic control	Well-established, licensed VLP vaccines (e.g., HBV, RSV)	High immunogenicity; proven expression of DENV1–4 VLPs; good safety	Due to their complex culture requirements, mammalian systems are typically the costliest. Production costs can reach up to ~$1,000 per gram of protein.	(Urakami et al., 2017; Vesikari et al., 2023; Fan et al., 2024; Sanchez-Martinez et al., 2024; Anumanthan et al., 2025).
Insect Cells (Baculovirus)	Sf9, Hi5, Bombyx mori	High (~mg to tens mg/L)	Shorter, mannose-rich glycans; efficient PTMs, furin-like cleavage	Highly scalable, medium cost, simpler culture conditions	Licensed vaccines (HPV, influenza, COVID-19); proven safety and efficacy	Successfully expressed dengue VLPs (DENV-1,4); robust immune responses; slightly altered glycosylation	Insect cell systems offer moderate costs (~$1.12/mg protein), balancing complexity and affordability.	(Cox and Hashimoto, 2011; Charoensri et al., 2014; Trombetta et al., 2022; Utomo et al., 2022; Hong et al., 2023; Anumanthan et al., 2025).
Yeast Systems	*Pichia pastoris, S. cerevisiae*	Very high (tens mg/L to g/L)	Mannose-rich, non-human glycosylation; can be humanized (GlycoSwitch)	Rapid, highly scalable; cost-effective; simple media	Decades of licensed VLP vaccines (HBV, HPV, malaria R21); strong regulatory familiarity	Expressed glycosylated dengue E protein; successful bivalent DENV1/2 VLPs; no ADE; cost-effective production	Yeast-based expression systems are highly cost-effective, typically ranging from $0.10 to $0.50 per mg of protein.	(Bazan et al., 2009; Mani et al., 2013; Tomé-Amat et al., 2014; Yun et al., 2014; Mariz et al., 2015; Feng et al., 2017; Shukla et al., 2018; Brachelente et al., 2023).
Plant-based Systems	*Nicotiana benthamiana*	Low (~2 µg/g leaf tissue)	Plant-specific glycosylation; humanization possible (glycoengineering); other PTMs (disulfide bonds, protease cleavage)	Potentially scalable; low-cost cultivation; purification challenges	Recently licensed COVID-19 VLP vaccine (Covifenz); emerging regulatory acceptance	Successfully expressed DENV-1 VLPs; immunogenic despite low yield; potential scalability pending improvements	Plant-based systems (e.g., Nicotiana benthamiana) offer extremely low-cost production, typically ranging from $0.01 to $0.10 per mg of protein.	(Ponndorf et al., 2021; Hager et al., 2022; Dhawan et al., 2023; Peralta-Cuevas et al., 2025).

VLP, Virus-like particle; PTM, post-translational modification; ADE, Antibody-dependent enhancement.

## Challenges in dengue VLP production and stability (enveloped vs. non-enveloped VLPs)

Due to their envelope-coated nature, dengue VLPs present persistent challenges in achieving high yields and stability. Production yields for dengue VLPs are typically low across expression systems. In mammalian cell culture, purified dengue VLP titers are often on the order of only a few milligrams per liter – for example, 293-F cells produced around 0.5–3 mg/L of VLP (depending on serotype) under standard conditions (Urakami et al., 2017), though optimized constructs have reached up to ~10 mg/L (Thoresen et al., 2024). In insect cell systems (e.g., baculovirus-infected or transiently transfected Sf9/S2 cells), yields are similarly in the low single-digit mg/L range in most reports (often requiring further optimization of signal sequences and processing enzymes to improve secretion) (Urakami et al., 2017; Thoresen et al., 2024). Yeast and plant-based systems have been explored, but with even lower outputs. *Pichia pastoris* can assemble dengue VLPs, but only at modest levels, and in *Nicotiana* plants, the purified VLP recovery was only on the order of micrograms per liter-equivalent (about 2 µg per gram of leaf tissue) (Ponndorf et al., 2021). These yields are below those of simpler non-enveloped VLPs like human papillomavirus (HPV). HPV L1 protein self-assembles efficiently; in comparable systems, it is not uncommon to obtain tens of mg/L of HPV VLPs (for instance, plant-made HPV chimeric VLPs can exceed 3 mg VLP per gram of leaf biomass (Mardanova et al., 2024), versus the ~2 µg/g achieved for dengue), reflecting how much more productive non-enveloped VLP expression can be. The requirement of dengue VLPs for co-expression of prM and E, proper furin cleavage, and budding through cellular membranes imposes a bottleneck on yield; many produced particles never correctly bud or are retained intracellularly, unlike HPV VLPs, which assemble spontaneously in the cytosol or nucleus and accumulate to high levels.

Envelope-containing dengue VLPs also exhibit poorer thermal and long-term stability than non-enveloped VLPs. Dengue VLPs are fragile, as heat or physical stress can disrupt the lipid membrane and E protein arrangement. In practice, dengue VLP immunogens often require storage at cold temperatures to maintain conformation, and even then, they can undergo aggregation or loss of epitopes over time. Notably, the folding and antigenicity of dengue VLPs are highly temperature-sensitive during production: VLPs produced at 31°C retain native E epitopes, whereas those produced at 37°C show disrupted epitopes and reduced immunogenicity (Boigard et al., 2018). This indicates a tendency of the dengue envelope proteins to misfold or rearrange at physiological temperature, impacting stability. By contrast, HPV VLPs (composed of the robust L1 capsid protein) are markedly more thermotolerant. The HPV L1 VLP vaccine is highly stable, with a measured half-life of approximately 130 months at 25°C; even at 37°C (body temperature), its potency half-life is 18 months. Differential scanning calorimetry of various HPV VLP types reveals melting temperatures often around 60–70°C, reflecting strong intermolecular contacts and disulfide bonds that hold the capsid together (Shank-Retzlaff et al., 2006). Such high thermal stability and long shelf-life are not reported for dengue VLPs, which lack comparably sturdy structural features. Indeed, envelope-coated flavivirus particles (and VLPs) tend to lose infectivity or antigenicity rapidly above ~40°C (Boigard et al., 2018), and formulation studies indicate they require careful buffering and cryoprotection for storage. No dengue VLP formulation to date has demonstrated the multi-year room-temperature stability seen with HPV vaccines.

Several structural and biochemical factors underlie why dengue envelope VLPs are less stable and more heterogeneous than non-enveloped VLPs. A key issue is the presence of the lipid bilayer itself: this envelope is prone to degradation. It can fuse or fragment, primarily upon freeze-thaw or agitation, leading to VLP aggregation. The lipid composition can markedly influence particle stability. One recent study showed that dengue VLPs are more stable when enriched in phospholipids, suggesting that specific host membrane compositions yield sturdier particles (Palur et al., 2025). Another challenge is the proper processing of the polyprotein: dengue VLP assembly requires cleavage of prM to M by furin in the trans-Golgi, and inefficient cleavage results in “immature” VLPs with uncleaved pr peptide. These immature particles have a different surface protein arrangement and often form heterogeneous, less stable structures that can aggregate or display altered antigenicity (Boigard et al., 2018). Even among fully mature VLPs, there can be variability in size and morphology, partly due to varying E protein content or incomplete icosahedral assembly. This heterogeneity contrasts with HPV VLPs, which form uniform icosahedral capsids ~55 nm in diameter with 360 copies of L1 and minimal variation. Furthermore, the dengue E protein is a class II fusion protein that must undergo complex conformational changes; if triggered (e.g., low pH or heat) on the VLP, it can cause particles to clump or collapse. Non-enveloped VLPs like HPV lack such metastable fusion-driven conformational states. Instead, HPV capsids are held together by stable protein-protein interfaces and require harsh conditions (e.g., high temperature or extreme pH) to dissociate, making them inherently more resistant to physical and chemical stress. In summary, the envelope-coated nature of dengue VLPs introduces fragility (due to the lipid membrane) and assembly complexity (requiring precise cleavage and folding), resulting in lower yields, greater particle heterogeneity, and inferior thermal/formulation stability relative to simpler non-enveloped VLPs such as HPV (Shank-Retzlaff et al., 2006; Boigard et al., 2018).

## Stability and formulation strategies for dengue VLP vaccines

The selection of an appropriate production platform also directly impacts the physicochemical and antigenic stability of the generated particles. Dengue VLPs must maintain their structural integrity and antigenic conformation during manufacturing, storage, and distribution. As enveloped particles with host-derived lipid membranes, dengue VLPs are inherently more sensitive to environmental stressors than non-enveloped VLPs​ (Gupta et al., 2023). Changes in temperature, pH, ionic strength, or shear stress can disrupt the ordered assembly of the 60 envelope (E) protein homodimers on the VLP surface, leading to aggregation or loss of native epitope presentation (Roldão et al., 2012; Samandoulgou et al., 2015). Thermal stability is a particular concern because dengue E proteins undergo conformational transitions at around 37–40°C that can alter quaternary structure. This contrasts with some related flavivirus VLPs (e.g., Zika), which exhibit better intrinsic heat stability (Pindi et al., 2020). Indeed, modeling and cryo-EM studies have shown that dengue VLPs are heterogeneous in size and undergo maturation-associated conformational changes, underscoring the need to stabilize their structure. Optimal lipid content in the VLP membrane has been identified as a key factor for stability – too few or too many lipid molecules can reduce particle rigidity​. Likewise, strong protein–lipid interactions help maintain the E/M protein lattice, and altering the membrane composition can modulate VLP stability (Raghuvamsi Palur et al., 2023). Antigenic stability refers to preserving the critical neutralizing epitopes on E proteins. If VLPs dissociate or misfold, important quaternary epitopes (such as those spanning E dimers) may be lost, reducing the vaccine’s effectiveness. For example, researchers have engineered dengue VLPs to adopt a “mature” conformation that exposes otherwise cryptic neutralization epitopes; these stabilized VLPs showed an improved breadth of antibody responses​ (Shen et al., 2018). Conversely, including specific viral components can affect antigenic quality: incorporating the precursor membrane (prM) protein can yield immature forms with different epitopes. Some dengue VLP vaccines intentionally omit prM to avoid inducing non-neutralizing antibodies that could contribute to enhancement​ (Rothen et al., 2024).

**Figure 5:**
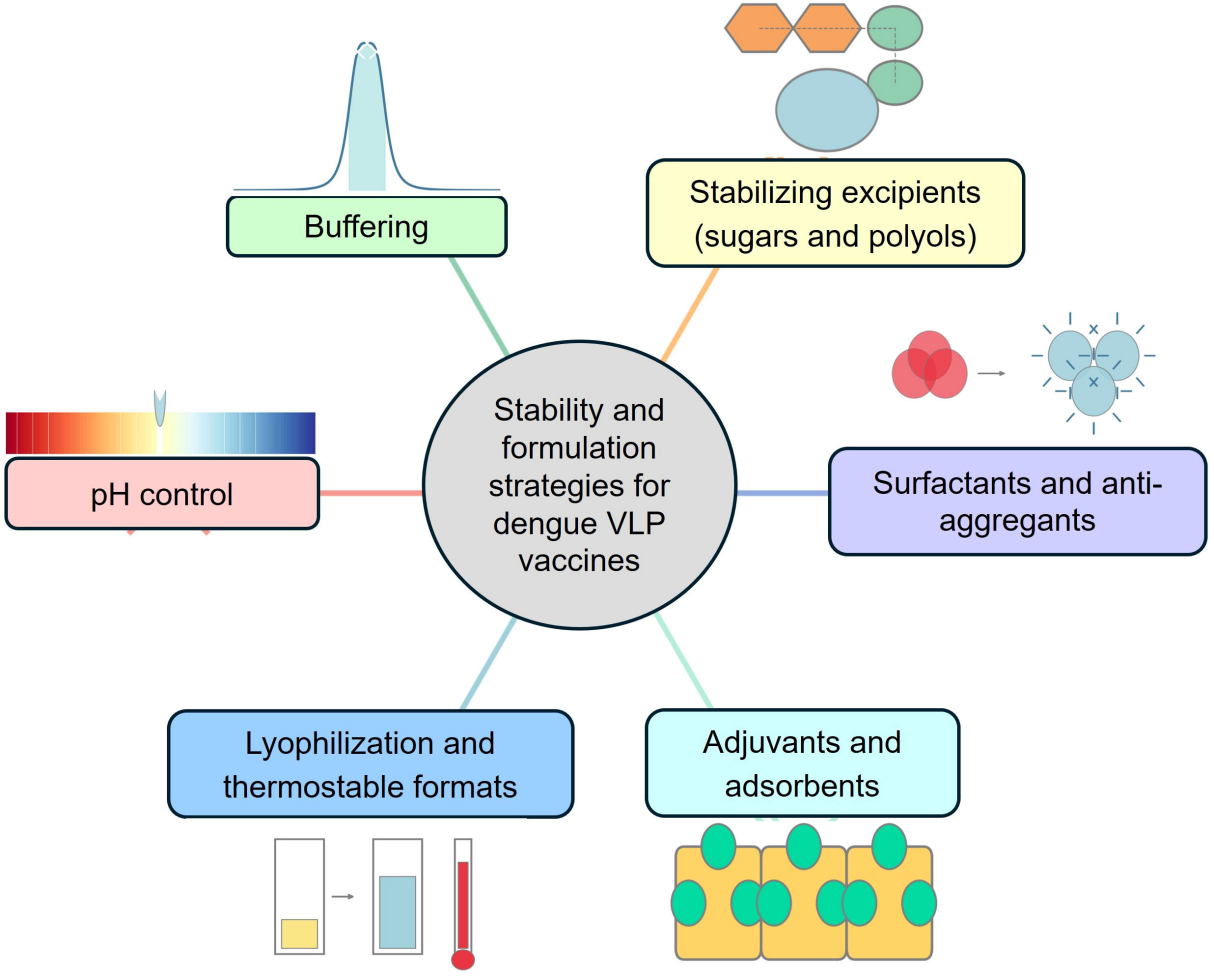
Schematic overview of stability and formulation strategies for dengue VLP vaccines. Key formulation approaches include pH control, buffering systems, stabilizing excipients (sugars and polyols), surfactants and anti-aggregants, adjuvants and adsorbents, and lyophilization to produce thermostable vaccine formats, which collectively ensure VLP structural integrity, antigen stability, and vaccine efficacy.

To ensure dengue VLPs remain stable and immunogenic over time, vaccine developers are adopting formulation approaches informed by both VLP biophysics and the success of licensed VLP vaccines (e.g., for hepatitis B and HPV). Key strategies are shown in **Figure 5** and include:

### Buffering and pH control

Dengue VLPs are formulated in buffered solutions (e.g., phosphate, histidine buffers) to maintain a neutral pH that preserves E protein dimer structure. For instance, a recent tetravalent dengue VLP candidate was formulated in a phosphate buffer with 5% sucrose (pH 7.2)​ (Thoresen et al., 2024). Histidine and borate buffers are used in HPV VLP vaccines (Gardasil^®^) to stabilize capsid proteins (Mejía-Méndez et al., 2022). Maintaining optimal ionic strength and osmolarity prevents conformational changes and avoids aggregation during storage​ (Fenner et al., 1987).

### Stabilizing excipients (sugars and polyols)

Lyophilization (freeze-drying) or freezing of VLPs requires cryoprotectants to prevent damage to the viral envelope. Sugars such as sucrose or trehalose replace water molecules and form an amorphous glass upon drying, which protects the VLP structure. In the dengue VLP formulation mentioned above, 5% sucrose was included to enhance thermal stability​ (Thoresen et al., 2024). Generally, adding 5–10% sugar during lyophilization significantly improves VLP recovery and thermostability​. This approach is widely used for viral vaccines distributed to tropical regions lacking robust cold chains (Gupta et al., 2023). Polyols (glycerol, sorbitol) and amino acids (glycine, arginine) can also stabilize proteins and prevent aggregation in solution and have been evaluated in VLP formulations (though specific reports for dengue VLPs are still emerging).

### Surfactants and anti-aggregants

Enveloped VLPs tend to aggregate or fuse, especially at high concentrations or upon agitation. Non-ionic surfactants like Polysorbate 80 are commonly added at low levels to VLP vaccines to prevent surface adsorption and aggregation. Polysorbate 80 has been used in hepatitis B vaccines (e.g., Engerix-B^®^) to maintain the dispersion of HBsAg VLPs​ (Grzelczak et al., 2010) and similarly will help dengue VLPs remain monodisperse. By embedding in the lipid envelope, surfactants can protect against shear stress during vial shaking or transportation. One stability study of a multivalent HPV VLP vaccine, including Polysorbate 80 and aluminum adjuvant, allowed the formulation to withstand extended light exposure and agitation without loss of potency​ (Liu, 2003).

### Adjuvants and adsorbents

Aluminum-based adjuvants (alum) boost the immune response and aid stability by adsorbing VLPs onto a particulate substrate. Dengue VLPs have been formulated with alum, as in a recent macaque study where the VLPs were mixed with aluminum hydroxide before injection (Thoresen et al., 2024). Adsorption to alum can shield VLPs from aggregation and proteolysis in the vial, although care must be taken as desorption kinetics and structural integrity on alum need to be optimized. Some licensed VLP vaccines use alum for immunogenicity and stability, e.g., HPV VLP vaccines adsorbed on alum retain conformational epitopes and have multi-year shelf stability at 2–8°C. Alum’s antigen binding can also reduce interface-induced degradation (e.g., preventing VLPs from sticking to vial walls). In addition, certain preservatives (like 2-phenoxyethanol, used in HBV vaccines) may be included to avoid microbial growth in multi-dose formulations without harming VLP structure (Lua et al., 2014).

### Lyophilization and thermostable formats

Lyophilized dengue VLP vaccines are being explored to alleviate cold-chain requirements. Freeze-drying VLPs with protectants (sugars, amino acids, polymers) allows the vaccine to be stored at ambient temperatures and reconstituted before use. This strategy has proven effective for other VLP-based vaccines – for example, experimental Ebola VLP formulations were lyophilized to improve thermostability for tropical deployment​ (Ye et al., 2006; Gupta et al., 2023). In HPV vaccines, research has shown that lyophilized or spray-dried VLPs can retain immunogenicity and remain stable at higher temperatures​ (Tumban et al., 2015). A lyophilized dengue VLP vaccine could tolerate excursions to 25–30°C. Accelerated stability studies provide encouraging data: a recombinant 9-valent HPV VLP vaccine maintained stability for *at least* 12 months at 25°C (and >72 months at 5°C) without significant loss of potency​ (Liu et al., 2025).

With proper formulation, including optimized buffers, stabilizers, surfactants, adjuvants, and preservation techniques, dengue VLP vaccines can achieve the physicochemical and antigenic stability necessary for real-world deployment, even in resource-limited settings. Drawing from proven formulation strategies in licensed VLP vaccines, these candidates can be engineered for long-term storage and global distribution with preserved immunogenicity and structural integrity.

## How do VLP-based dengue vaccines compare with other vaccine platforms?

VLP-based dengue vaccines induce balanced neutralizing antibody (NAb) responses against all four DENV serotypes, demonstrating consistent geometric mean titers (GMTs) across serotypes within ~5-fold ranges and maintaining seropositivity for at least one year in non-human primates (NHPs) (Metz et al., 2015). This balanced response contrasts sharply with live-attenuated vaccines such as Dengvaxia (CYD-TDV), which exhibit serotype-dependent imbalances, notably weaker responses and efficacy against DENV-1 compared to DENV-4, leading to increased risks of ADE in seronegative recipients (Sabchareon et al., 2012; Sridhar et al., 2018). Similarly, TAK-003 vaccine efficacy demonstrates some variability across serotypes, although it is generally improved compared to Dengvaxia (Biswal et al., 2020).

Neutralizing antibody magnitudes vary significantly among platforms. **Table 2** shows these comparative evaluations, highlighting differences in immunogenicity, durability, stability, safety, and production considerations across dengue vaccine platforms. For instance, mRNA vaccines achieve exceptionally high neutralizing titers (peak PRNT_50_ around 10³–10^4^ in mice), suggesting potential robust immunity comparable to natural infection (Wollner et al., 2021). This is because the antigen is produced *in situ* by host cells, yielding proteins that fold and assemble with native post-translational modifications into authentic VLPs, thus displaying critical conformational epitopes (including quaternary E protein structures) exactly as in the live virus (De Lorenzo et al., 2020). Each mRNA-transfected cell can produce many copies of the antigen over an extended period, effectively prolonging antigen presence compared to a one-time protein inoculation. Furthermore, mRNA-LNP vaccines are inherently self-adjuvanting, the delivered mRNA triggers innate immune sensors (e.g., RIG-I, MDA5, TLR7/8), causing local release of inflammatory cytokines/chemokines and activation of dendritic cells and other antigen-presenting cells (Edwards et al., 2017; Zhang et al., 2020).

**Table 2:** Comparative evaluation of dengue vaccine platforms: immunogenicity, durability, stability, safety, and production considerations.

CYD-TDV (Dengvaxia) *Live-attenuated chimeric (YF17D backbone)*	Induces neutralizing antibodies against all four serotypes after three doses, but overall efficacy (~60%) is moderate, with weaker responses to DENV-1 and DENV-2. T-cell responses are reported but limited.	Antibodies last ~5 years in seropositive individuals; protection declines by ~30 months in seronegative recipients, increasing illness risk.	Lyophilized; stable 3 years at 2–8°C. Requires reconstitution; use within 30 min. Needs cold-chain storage.	It is well-tolerated (low reactogenicity). ADE risk in seronegative individuals is increasing severe dengue hospitalizations. There have been no short-term serious adverse events. It is restricted to seropositive individuals.	Produced in Vero cells as four separate chimeric viruses, making the serotype balancing complex. The three-dose schedule (~$20/dose, ~$60 total) increases logistics and costs—standard refrigeration required.	Licensed in 2015 (first dengue vaccine), approved in many endemic countries and by FDA (2019) for ages ≥9 with prior dengue exposure.	(Sabchareon et al., 2012; Dayan et al., 2013; Sridhar et al., 2018; Coudeville et al., 2020)
TAK-003 (Takeda) *Live-attenuated tetravalent (DENV-2 backbone)*	Induces robust neutralizing antibodies after two doses (3 months apart), strongest for DENV-2 (vaccine backbone) and DENV-1; lower but still substantial for DENV-3 (≥88% seropositive at 3 years) and DENV-4. Elicits dengue-specific CD4^+^ and CD8^+^ T-cell responses.	Neutralizing antibodies persist ≥3 years (36 months: ~97–99% seropositive DENV-1/2, ~88% DENV-3, ~56% DENV-4). Efficacy ~80% first year, ~54% by year 3, showing some decline (mainly DENV-3/4). Protection against severe disease remains strong at 4–4.5 years.	Liquid formulation: stable at 2–8°C (no ultra-cold storage required). Ready-to-use; no reconstitution is needed. Stable for months under refrigeration.	Well tolerated; no serious vaccine-related adverse events in Phase 3 (~4.0% SAE vs. 4.8% placebo). No dengue enhancement in seronegatives. Common mild side effects (injection-site pain, fever) are similar to those of other pediatric vaccines.	Produced as four live viruses (one attenuated DENV-2, three chimeric DENV-2/1,3,4) in cell culture. A common backbone simplifies manufacturing. Compared to Dengvaxia, competitive pricing is anticipated for endemic regions, and the two-dose regimen reduces cost and logistics.	Phase 3 was completed (~20,000 children). It has been approved in the EU (2022) and Indonesia (ages 4–60). Regulatory submissions are ongoing elsewhere.	(ClinicalTrials.gov, 2016; ClinicalTrials.gov, 2018; ClinicalTrials.gov, 2018; Biswal et al., 2020; Turner et al., 2020; Sirivichayakul et al., 2022)
TV005 (NIAID/Butantan) *Live-attenuated tetravalent (rDEN1–4 mix)*	A single dose elicits strong tetravalent immunity. Human challenge study: 100% protection against DENV-2/3 at 6 months; placebo group fully infected. ~90% seroconversion to all four serotypes in prior trials. Robust T-cell responses (e.g., IFN-γ) were also observed.	A single dose induces durable immunity; neutralizing antibodies persist 1–2 years. In challenge studies, sterilizing protection lasts 6 months. Phase 3 (Butantan-DV) shows efficacy lasting ~3–4 years (especially DENV-1/2). Durability beyond 5 years is ongoing.	Lyophilized vaccine stable at 2–8°C; long shelf-life refrigerated and reconstituted before use. In-house production by Butantan Institute ensures a stable tropical supply.	Excellent safety; no serious vaccine-related AEs. Mild reactions (low-grade fever, injection-site redness) were comparable to placebo. No dengue-like illness or ADE observed. Safe for dengue-naïve Individuals.	Produced in Vero cells as four attenuated strains (three 3′UTR deletion mutants, one DENV-2 chimera). Freeze-dried, single-dose vial reduces distribution costs. Expected low-cost for public health (e.g., India's Phase 3 "DengiAll").	Phase 3 ongoing (~17,000 subjects in Brazil; Indian trial by Panacea/ICMR). Not yet licensed; leading candidate pending efficacy results.	(Mohanty et al., 2022; Nogueira et al., 2024; Pierce et al., 2024)
VLP Vaccine (DENVLP) *VLP (tetravalent subunit)*	Induced high neutralizing antibodies to all four DENV serotypes in mice and NHPs (100% seroconversion). High, balanced titers in macaques, no antibody interference between serotypes. Primarily CD4^+^ T-helper responses; alum adjuvant enhances robust antibody production.	Protective neutralizing antibodies persisted ≥12 months in macaques (all remained seropositive). Durability comparable to live vaccines in animals. Long-term human durability unknown; related flavivirus VLP vaccines show multi-year persistence.	Non-replicating subunit; stable at 2–8°C, no viral spread risk. Expected shelf life ≥1 year, suitable for lyophilization. No ultra-cold storage required.	Non-live, inherently safe; cannot cause dengue. No adverse effects or *in vitro* ADE observed in preclinical tests. Mild injection-site inflammation was typical of protein vaccines. Suitable for children and immunocompromised individuals.	Produced via recombinant expression of prM/E proteins in cell culture, this vaccine is complex in its purification process but leverages existing subunit vaccine technology. It has a moderate-to-high cost per dose, but adjuvants reduce dosage. No high-level biocontainment is needed; it is rapidly scalable and adaptable.	Preclinical stage; immunogenic and protective in mice and monkeys. Phase 1 trial planned based on promising NHP results.	(Thoresen et al., 2024)
mRNA-LNP Vaccine (e.g. prM/E mRNA) *Nucleoside-modified mRNA in lipid nanoparticle*	DENV mRNA vaccine (prM/E) is highly immunogenic in mice; robust neutralizing antibodies and virus-specific T-cell responses comparable to live infection. Fully protected mice (AG129) from lethal DENV challenge. Serotype-specific antibodies with minimal cross-reactivity, reducing ADE risk.	Long-term human durability is unknown (no clinical data yet). Protective antibodies persisted for several weeks post-boost in mice. Based on other flavivirus mRNA vaccines, boosters are likely needed and rapidly reformulated if immunity declines.	Requires cold-chain storage; stable months at –20°C, shorter at 2–8°C. No live components: freeze–thaw affects mRNA integrity. Advanced stabilization (lyophilization/next-gen LNPs) is possible. Handling is similar to that of COVID-19 mRNA vaccines.	Non-infectious, well-tolerated in animals; no dengue risk, mild transient inflammation in mice. Human reactogenicity is expected to be similar to other mRNA vaccines (transient fever, injection-site pain); no serious preclinical concerns. ADE risk minimized; no *in vitro* enhancement observed.	Rapid, scalable *in vitro* transcription (no cell culture). A multivalent vaccine is made by mixing monovalent mRNAs. Cost decreases with scale (~$2–3/dose for COVID-19). LNP formulation and cold chain add costs. No live virus or yeast simplifies production and quality control.	Preclinical proof-of-concept demonstrated in mice (protective in challenge studies). The tetravalent mRNA vaccine is under development; there are no clinical trials yet. Ready for Phase 1, supported by mRNA successes in other diseases.	(Wollner et al., 2021)
DNA Vaccine (TVDV) *Plasmid DNA (tetravalent prM/E)*	Early tetravalent DNA vaccine (TVDV) showed poor antibody but strong T-cell responses. Phase 1 (with Vaxfectin): few neutralizing antibodies, but ~80% had robust IFN–γ T-cell responses at high dose.	Antibody responses are low and short-lived (wane by 6–12 months, many never seropositive). T-cell memory was durable and recallable, but without sufficient antibodies, protection was inadequate. Unmodified DNA vaccine alone provided limited durability.	DNA is highly stable at ambient and refrigerated temperatures (years if dried or frozen). Standard refrigeration (2–8°C) is sufficient for TVDV. High stability is advantageous for tropical storage.	Excellent short-term safety, no serious AEs, only mild injection-site pain. No dengue-like illness (non-live vaccine). Theoretical ADE risk not observed clinically. Safe but ineffective.	Easy, low-cost manufacturing in *E. coli*. No biosafety hazards. Formulated with lipid adjuvant (Vaxfectin). Requires multiple high doses or electroporation to improve efficacy, adding complexity. The three-dose schedule is shorter than live vaccines, but insufficient immunogenicity limits practical use.	Phase 1 was completed (U.S., 2018); it was safe but had low immunogenicity. No further trials; improved next-gen DNA vaccines in development	(Danko et al., 2018)
Adenovirus-Vectored Vaccine *Viral vector (Ad5-DENV2-NS1/EDIII)+boost*	Preclinical adenoviral vaccine induced potent antibody and T-cell responses. Ad5-based DENV-2 (EDIII, NS1) vaccine elicited high neutralizing antibodies in mice, enhanced by prime–boost. Balanced Th1/Th2, strong IFN–γ T-cell response. Successful multivalent (prM/E tetravalent) tests in mice.	Limited durability data (~2 months post-boost in mice). Prime boost establishes memory B/T cells. Adenovirus vectors typically induce lasting T-cell memory; antibody longevity requires further studies. Boosters are likely needed for long-term protection.	Adenoviral vaccines are stable at 2–8°C (weeks; ~1 year lyophilized). There is no live dengue virus; a standard cold chain is sufficient, and no deep freeze is required.	Replication-defective adenovirus was safe, minor transient reactions in animals, no dengue risk. Pre-existing anti-Ad5 immunity may reduce efficacy. No ADE observed in mice; balanced immunity reduces ADE risk.	Produced via adenovirus culture (HEK293 cells), purified using established methods (e.g., COVID-19 vaccines). Scalable but costlier than plasmids; tetravalent mixes add complexity. Faster than live-virus vaccines; thermostable liquid.	Preclinical – tested in mice (and some NHP experiments). No human trials of a tetravalent Ad dengue vaccine have been reported yet. (Related vector approaches for dengue, e.g., measles-virus-vectored, are also in preclinical stages.) The promising mouse immunogenicity results​ support further development, potentially toward Phase 1.	(Shoushtari et al., 2025)

VLP and adjuvanted inactivated vaccines consistently induce high titers (GMT >1,000 in animal studies). A heterologous prime–boost regimen (mRNA prime followed by VLP boost) leverages the complementary strengths of each platform to maximize immune protection. Priming with an mRNA vaccine induces a broad immune response, including substantial CD4^+^/CD8^+^ T cell activation and baseline neutralizing antibody production, owing to the antigen being produced inside cells and presented on MHC molecules. However, a booster with a purified VLP (protein-based) can powerfully restimulate B cells and significantly amplify neutralizing antibody titers. The VLP presents a high-density array of viral epitopes to the immune system, and when given after an mRNA prime, it can recall and expand memory B cells to produce a rapid surge in high-affinity antibodies. Importantly, this strategy can yield a more balanced and durable immunity by combining mRNA’s strong T-cell priming with the robust antibody boosting of a protein VLP (De Lorenzo et al., 2020). Evidence from flavivirus models supports the feasibility of such heterologous approaches. In a Zika virus study, a vector-based prime followed by a VLP boost led to significantly higher neutralizing-antibody levels than the prime alone, while maintaining the T-cell response from the priming dose (De Lorenzo et al., 2020). Similarly, a West Nile virus DNA-prime/protein-boost regimen in mice showed that although the DNA alone elicited little antibody, the subsequent protein boost elicited a marked rise in neutralizing antibodies and conferred protection against lethal challenge (De Filette et al., 2014). In dengue models, combining different vaccine platforms has proven synergistic: for example, a DNA vaccine prime with a viral replicon particle boost in nonhuman primates produced the highest DENV-1 neutralizing titers and completely prevented viremia upon challenge, outperforming either modality alone (Valdés et al., 2019). While clinical data on mRNA-prime/VLP-boost in dengue are still forthcoming, these preclinical findings address the reviewer’s concern by demonstrating a sound immunological rationale. The heterologous mRNA prime–VLP boost strategy is scientifically well-founded and likely to enhance overall vaccine efficacy by marrying the potent cellular immunity from the mRNA prime with the heightened humoral response from the VLP boost, thereby combining the advantages of both platforms in a single regimen.

In contrast, DNA vaccines show lower antibody responses even with adjuvant, remaining below protective thresholds in human trials but exhibiting robust T-cell responses. For instance, in a Phase 1 trial of a tetravalent dengue DNA vaccine (formulated with a cationic lipid adjuvant), a 1 mg dose elicited no detectable neutralizing antibodies and a 2 mg dose induced only minimal antibody titers, whereas robust IFNγ-secreting T-cell responses were observed in 50–79% of vaccine recipients (Danko et al., 2018). Similarly, an earlier monovalent DENV-1 DNA vaccine required a high 5 mg dose to induce any measurable neutralizing antibodies (only ~42% of subjects seroconverted, vs. 0% at 1 mg), despite eliciting T-cell responses in >80% of high-dose recipients (Beckett et al., 2011). These findings underscore the limited antibody immunogenicity of dengue DNA vaccines even when adjuvanted, in contrast to their consistently strong cellular immunity. Several platform-specific factors likely contribute to this outcome: the *in vivo* antigen expression from plasmid DNA is relatively inefficient, necessitating high doses and optimized delivery methods for robust B-cell responses. Indeed, while adding Vaxfectin^®^ adjuvant markedly enhanced neutralizing titers and protection in a nonhuman primate dengue DNA vaccine study, the same formulation in humans yielded only subprotective antibody levels, partly due to dose limitations (the Phase 1 trial’s 2 mg maximum was constrained by formulation chemistry). Moreover, conventional intramuscular needle delivery may not transfect sufficient cells; alternative delivery technologies such as jet injectors or electroporation have shown improved immunogenicity by facilitating plasmid uptake (Danko et al., 2018). Finally, the antigen design itself, typically plasmids encoding the dengue prM and E glycoproteins, must achieve efficient expression and proper folding (e.g., assembly of virus-like particles) to stimulate potent neutralizing antibodies, which remains a challenge for the DNA platform. As a result, dengue DNA vaccines to date have not achieved the robust humoral responses seen with other vaccine platforms, despite their favorable T-cell profiles and safety.

Regarding durability, live-attenuated vaccines provide extended antibody persistence (~80% efficacy first year, ~54% by third year with TAK-003) (Biswal et al., 2020). In contrast, protein subunit vaccines show rapid antibody waning, necessitating booster formulations (Manoff et al., 2019). VLP vaccines demonstrate intermediate antibody durability, with macaques maintaining protective titers for ≥12 months without significant decline (Metz et al., 2015). Regarding cellular immune responses, live vaccines such as TAK-003 induce comprehensive and multifunctional CD4^+^ and CD8^+^ T-cell responses detectable for ≥3 years post-vaccination (~80–90% positivity) (Biswal et al., 2020). mRNA vaccines also stimulate significant T-cell responses in preclinical models (Wollner et al., 2021). VLP vaccines predominantly elicit CD4^+^ T-helper responses essential for antibody production; however, detailed quantitative data on CD8^+^ responses remain limited (Metz et al., 2015). Despite low antibody production, DNA vaccines notably enhance T-cell responses (~79% IFN-γ^+^ T-cell positivity in Phase 1 trials) (Danko et al., 2018).

Safety profiles favor non-replicating platforms (VLP, mRNA, DNA, adenoviral vector) due to inherently lower ADE risks. Clinical and preclinical trials of non-live vaccines consistently report mild reactogenicity and no serious adverse events (Metz et al., 2015; Danko et al., 2018; Wollner et al., 2021). In contrast, live vaccines involve transient viral replication, mild systemic symptoms, and potential ADE risk, particularly in dengue-naïve individuals (Sridhar et al., 2018).

Manufacturing and stability vary significantly across platforms. Live vaccines necessitate careful virus attenuation, serotype balancing, and cold-chain storage. VLP vaccines benefit from recombinant expression systems, optimized formulations (e.g., lyophilization with cryoprotectants such as sucrose), and stable storage at 2–8°C (Metz et al., 2015). mRNA platforms offer rapid production but require stringent storage (–20°C), while DNA and viral vector vaccines offer practical stability under standard refrigeration (Danko et al., 2018).

In summary, VLP-based dengue vaccines uniquely combine balanced and robust antibody responses, favorable durability, inherent safety, and feasible production, positioning them as a promising candidate for next-generation dengue prevention.

## Future perspectives in VLP-based dengue vaccines

The development of multivalent dengue VLP vaccines represents a promising and realistic pathway to achieving comprehensive and balanced protection against all four dengue virus serotypes. Recent preclinical research demonstrates that tetravalent VLP formulations elicit robust neutralizing antibodies across all dengue serotypes without triggering ADE, with neutralizing antibody titers consistently reaching GMTs >1,000 across serotypes in animal models (Rothen et al., 2024; Thoresen et al., 2024). Advanced techniques such as mosaic particle designs and engineered epitope presentations could further optimize broad-spectrum immunity and streamline production processes, although empirical validation in clinical studies remains essential (Shen et al., 2018; Rothen et al., 2024).

Integrating innovative adjuvants and delivery technologies represents another critical dimension for enhancing VLP vaccine efficacy. Potent immunostimulants, including saponin-based adjuvants (e.g., QS-21) and Toll-like receptor (TLR) agonists, have shown promising results in preclinical trials by significantly enhancing neutralizing antibody titers (up to 5-fold increases) and promoting favorable Th1-biased immune responses (Rothen et al., 2024). Additionally, mucosal vaccine delivery methods (intranasal or oral) offer an attractive route for inducing mucosal immunity, improving vaccine accessibility, and enhancing public acceptance. However, robust comparative efficacy data against parenteral delivery remain necessary to validate these benefits in humans (Gupta et al., 2023).

Ensuring vaccine thermostability is vital, especially considering the tropical climates of dengue-endemic regions. Recent technological advancements in lyophilization and thermostable formulations, such as microarray patches and spray-dried vaccines, have successfully preserved VLP immunogenicity at elevated temperatures (up to 40°C for several months), significantly minimizing cold-chain dependence and logistical barriers (Tumban et al., 2015; Gupta et al., 2023).

Nonetheless, substantial regulatory and logistical hurdles remain. Demonstrating long-term safety and balanced immunogenicity across diverse populations remains challenging, particularly given past concerns associated with previous dengue vaccines such as Dengvaxia. The absence of universally accepted correlates of protection complicates regulatory pathways, necessitating lengthy and costly efficacy trials (Sridhar et al., 2018). Moreover, region-specific infrastructure limitations, economic constraints, and variable healthcare delivery capacities, particularly in Latin America and Southeast Asia, pose additional obstacles.

We propose several actionable strategies to navigate these challenges: a) Platform harmonization: establish standardized, modular VLP production platforms validated by preclinical and early clinical benchmarks, facilitating rapid regulatory review and efficient scale-up. b) regional manufacturing hubs: develop local manufacturing capabilities in endemic regions to shorten supply chains, enhance vaccine availability, and foster regulatory confidence and community acceptance. c) Adaptive regulatory frameworks: employ adaptive clinical trial designs and explore validated surrogate endpoints, such as neutralizing antibody thresholds correlated with protection, to expedite regulatory approval processes. d) Global coordination and financing: strengthen international collaboration for vaccine procurement, including advanced market commitments and pooled funding mechanisms, with clearly defined governance and financial commitments to ensure equitable distribution and affordability. By integrating these scientific innovations and pragmatic deployment strategies, dengue VLP vaccines have the potential to become a globally impactful solution. Future research and coordinated global efforts should prioritize overcoming these critical barriers, ultimately translating promising preclinical findings into tangible public health benefits.

## Conclusions

In summary, dengue VLP vaccines offer a next-generation approach with clear advantages over live-attenuated dengue vaccines. VLPs are non-replicating and can be precisely engineered to include purified dengue antigens of all four serotypes, minimizing the safety risks associated with live vaccines​. This controlled composition avoids the replicative interference seen in tetravalent live formulations, enabling a more balanced neutralizing immunity against each DENV serotype while reducing the potential ADE. Moreover, VLP platforms have an excellent safety and immunogenicity record in humans (e.g., hepatitis B and HPV vaccines), underscoring their proven tolerability across populations. These attributes, high safety, balanced multivalent immune responses, and tight antigenic control, position dengue VLP vaccines as an attractive and potentially superior alternative to traditional live‐attenuated approaches.

Recent preclinical advances have reinforced the feasibility of multivalent dengue VLP designs and paved the way for clinical translation. Notably, a tetravalent DENV VLP vaccine elicited robust neutralizing antibodies to all four serotypes in 100% of immunized non-human primates for at least one year, with no signs of ADE observed *in vitro*. Likewise, novel VLP constructs in mice have induced high-affinity, serotype-spanning antibody responses without generating enhancing antibodies​. However, the strong claims of balanced immunity and absence of ADE observed in these promising non-human primate studies must be interpreted cautiously, as translating preclinical findings into robust clinical efficacy and safety in humans remains a significant challenge. It is important to recognize that NHP models might not fully replicate human ADE mechanisms or immune dynamics, emphasizing the necessity of thorough clinical evaluation. Building on this encouraging groundwork, a strong rationale exists to advance dengue VLP candidates into clinical development. Key priorities will include optimizing formulation stability (some dengue VLPs remain stable for extended periods at 4°C, facilitating storage and distribution​) and scaling up manufacturing using modern recombinant production systems to ensure supply meets global demand. Regulatory pathways should also be navigated early, leveraging the established precedent of VLP-based vaccines to expedite approval. Finally, realizing the public health impact of dengue VLP vaccines will require coordinated international investment and capacity-building. This includes bolstering regional manufacturing in dengue-endemic areas and adopting adaptive regulatory strategies to accelerate vaccine evaluation and deployment. Such coordinated efforts are essential to bring these innovative VLP platforms from bench to bedside, fulfilling their promise of safe, effective, and broadly protective dengue vaccination.
